# The Repair of Furcal Perforations in Different Diameters with Biodentine, MTA, and IRM Repair Materials: A Laboratory Study Using an* E. Faecalis* Leakage Model

**DOI:** 10.1155/2018/5478796

**Published:** 2018-01-15

**Authors:** E. Övsay, R. F. Kaptan, F. Şahin

**Affiliations:** ^1^Department of Endodontics, Faculty of Dentistry, Yeditepe University, İstanbul, Turkey; ^2^Department of Genetics and Bioengineering, Faculty of Dentistry, Yeditepe University, İstanbul, Turkey

## Abstract

The aim of this study is to evaluate the microleakage of repair materials MTA, IRM, and Biodentine applied on furcal perforations with different diameters. One hundred and forty extracted human teeth were used in this study. The teeth were divided into 2 main groups (60 teeth in each) which were then divided into 3 subgroups (*n* = 20). The remaining 20 teeth were divided into 2 groups (10 in each) to serve as controls. The furcal areas of the teeth were perforated with #2 cylindrical burs in Group 1 whereas perforations were made using #4 cylindrical burs in Group 2. Each subgroup of both Groups 1 and 2 received ProRoot MTA (ProRoot, USA), Biodentine (Septodont), or IRM (Dentsply, USA) to repair the perforations. An experimental set-up was established to contaminate repaired perforations with* E. Faecalis* (ATCC29212). The turbidity of bacteria was observed on the 7th, 15th, 30th, and 45th days. The data was analysed by chi-square test (*p* > 0.05). The number of bacteria in the group perforated by bur #2 and closed by MTA was found to be lower than the other groups on the 7th day (*p* < 0.05). There was no statistical difference in the bacterial counts of other groups on the 15th, 30th, and 45th days (*p* > 0.05). ProRoot MTA was found to be more successful in the prevention of bacterial leakage compared to IRM and Biodentine in smaller perforations during the 1st week.

## 1. Introduction

 The primary aim of the root canal treatment is to prevent apical periodontitis which is a consequence of bacterial contamination within the root canal system [1]. Thus, the success of endodontic treatment is highly dependent on the prevention recontamination of root canal space following disinfection. Adequate shaping, irrigation, and hermetic seal of the root canal system are indispensable steps to achieve this goal.

Perforations are mishaps that might occur during the course of endodontic treatment mainly due to iatrogenic factors. However, they might also occur due to extensive decay of dentinal structure. A perforation creates a pathological passage between the root canal system and the periodontium and jeopardizes the success of the endodontic therapy. The damage caused by the perforation may eventually result in the extraction of the compromised tooth [2].

A wide range of materials were used to seal perforations such as amalgam, composite, zinc oxide eugenol, and Mineral Trioxide Aggregate (MTA). Mineral Trioxide Aggregate is a silicate based material containing various radiopacifiers depending on the brand. Its favourable features such as biocompatibility, induction of hard tissue deposition, and tissue healing and high pH renders it the material of choice for a variety of dental procedures [3, 4].

“Intermediate restorative material” (IRM, Caulk, Dentsply, Milford, DE) is a sealing material enforced with polymer including zinc oxide and eugenol. Its higher mechanical strength is one of the major reasons for the preference of IRM as a temporary restorative material [5].

Biodentine (Septodont, Saint Maur de Fossés, France) is another silicate based material having similar characteristics with MTA. According to the manufacturer, it provides a hermetic seal and durable restoration and it is advocated in reparative treatment procedures where an optimal sealing is expected. However, there are a limited number of studies assessing its sealing ability.

As perforations create pathological pathways enhancing the contamination of the periodontal space, it is logical to assume that the damage would be greater as the size of the perforation increases. In such situations, the sealing ability of the repair material would be the most significant factor determining the prognosis of the healing procedure.

A survey of the literature shows that there is yet no research focusing on the relation between bacterial microleakage and perforation size as well as the type of repair material [6]. The aim of this study was to evaluate the effect of perforation size, type of root repair material, and time on the amount of bacterial microleakage using an in vitro study design.

## 2. Materials and Methods

### 2.1. Tooth Preparation

This study was revised and approved by the Ethics Committee of Yeditepe Health Studies School of Medicine. One hundred and forty intact human maxillary and mandibular molars were used. The experimental teeth were extracted because of periodontal reasons. The teeth were examined under a 10x surgical microscope (Carl-Zeiss, Oberkochen, Germany) and those with similar anatomical characteristics and free of cracks were selected.

The teeth were autoclaved and kept in an ultrasonic bath (Bandelin – RK 100) filled with 2.5% sodium hypochlorite (NaOCl) for 10 minutes. Later, they were sectioned by a microtome (Anglia Scientific, Cambridge, UK) from the cementoenamel junction and the root sections were standardized to 16 mm by digital calliper. The teeth were kept in deionised water at 4°C until they were processed. The specimens were divided into two groups (*n* = 60). Furcal perforations having 2 mm and 4 mm diameters were created by using #2 and #4 cylindrical burs (Jota, Switzerland) (Table 1).

The root canal orifices and the apical ends of the roots were then sealed with cyanoacrylate adhesive (By-1500 Cyanoacrylate Adhesive, By Best, Ergin Industry, Turkey) in an attempt to increase the marginal seal.

### 2.2. Repair of Perforations

Distribution of perforations and repair materials among groups were as follows.

*Experimental Groups*

 
*Group 1-*MTA: perforation with size 2 bur + MTA
 
*Group 1-*BIOD: perforation with size 2 bur + Biodentine
 
*Group 1-*IRM: perforation with size 2 bur + IRM
 
*Group 2-*MTA: perforation with size 4 bur + MTA
 
*Group 2-*BIOD: perforation with size 4 bur + Biodentine
 
*Group 2-*IRM: perforation with size 4 bur + IRM

*Control Groups*

 
*1st positive control:* perforation with size 2 bur with no repair material applied
 
*2nd positive control:* perforated with 4 no bur with no repair material applied
 
*Negative control:* not perforated and no material applied

*In G1 and G2*, 1 g of MTA (Pro-Root MTA) was mixed according to the manufacturer's instructions, with 0.35 mL of distilled water to produce a homogeneous paste. An amalgamator was used for capsule preparation. The MTA was placed in the perforation with a MAP System (Dentsply, Maillefer, Switzerland) and compacted with Schilder pluggers (Hu Friedy, Chicago, IL, USA).

*In G1 and G2*, 1/1 powder liquid ratio was homogeneously prepared. IRM was mixed according to manufacturers' instructions and applied to the furcal areas with an amalgam carrier.

*In G1 and G2*, Biodentine was prepared with 5 drops of liquid placed into a special capsule and vibrated in an amalgamator for 30 seconds. The material was then applied with a hand carrier.

While applying the materials, pressure and extreme effort for condensation were avoided. Two coats of nail varnish were applied on the external surfaces of all teeth, except the area with 2 mm radius around the perforation. This was done to prevent bacterial leakage through lateral canals or other discontinuities in the cementum. All groups were kept in a 100% humid environment at 36°C for incubation until the second part of experimentation.

### 2.3. The Microleakage Set Up

**Figure 1:**
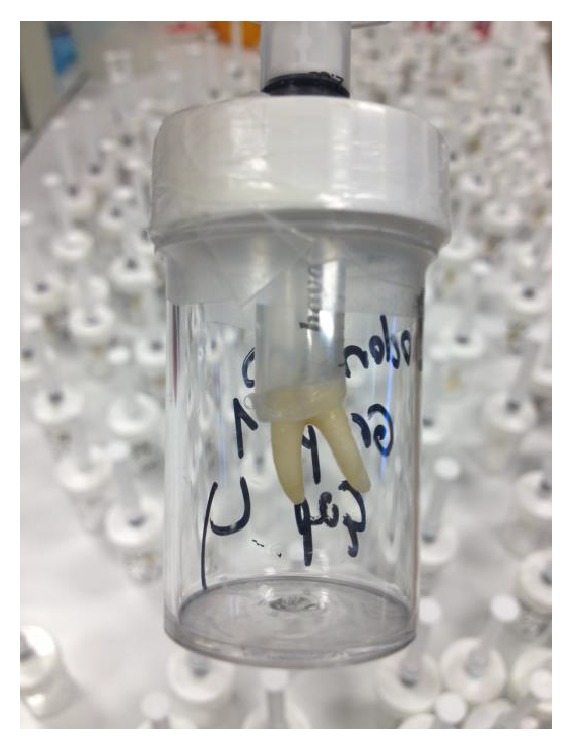
The microleakage model modified from Imura et al. (1997).

The experimental set-up used in this study was modified from the one described previously by Imura et al. (1997) (Figure 1).

Each specimen was inserted in a (0.5*∗*3) plastic needle shortened to gain tight junction between the specimen and the plastic needle. The plastic needle was used to create the bacterial reservoir. The interface between the tooth and the silicone hose was sealed with cyanoacrylate adhesive.

All teeth, plastic needles, and the hose were exposed to UV in a special cabin for 60 minutes to sterilize the equipment.

### 2.4. The Experimental Set Up

**Figure 2:**
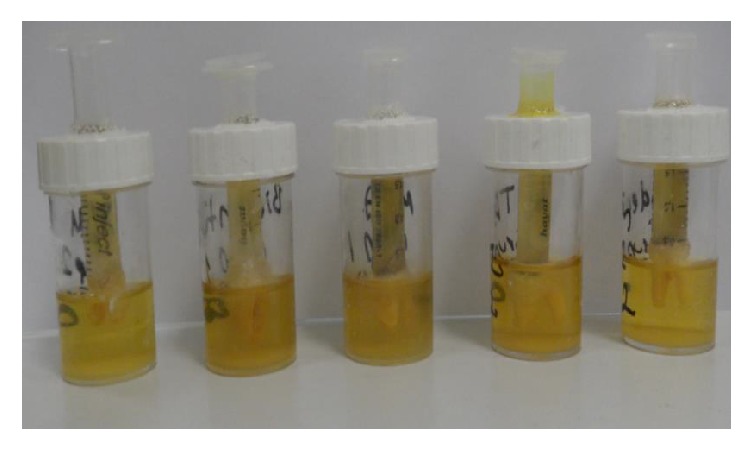
The blurred colour of the medium indicating the presence of contamination with* E. Faecalis*.

The teeth attached to the plastic needles were then inserted into the plastic hub and sealed with cyanoacrylate adhesive. The reservoirs were filled with* E. faecalis.* An* E. faecalis* specific medium (Azide Dextrose Broth, (Oxoid, Ogdensburg, New York, USA.) was placed into the hub to detect the leakage. All these processes were performed in a sterile cabin to prevent the contamination. The specimens were observed on a daily basis and data were collected on days 10, 15, 30, and 45. Any turbidity in the medium was recorded as leakage (Figure 2).

**Figure 3:**
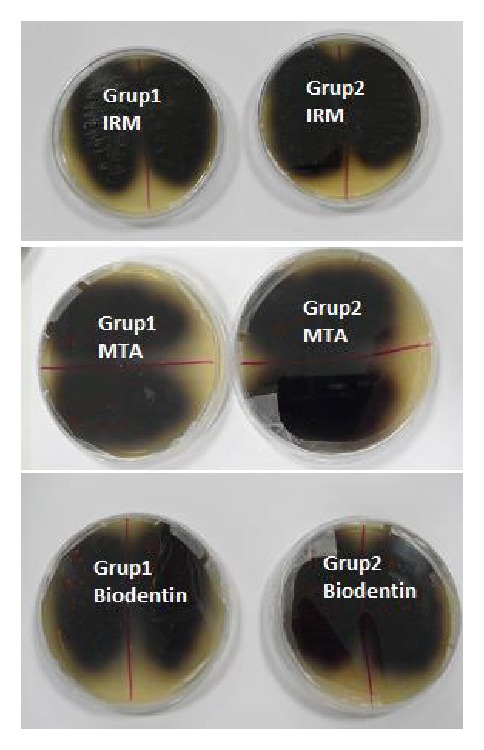
Agar experimentation showing that all specimens were contaminated by* E. Faecalis*.

The leaked samples were carried into the* E*.* Faecalis* specific Bile Aesculin Azide Agar (Bile Aesculin Azid Agar, Merk, KgaA, Darmstad, Germany) to determine if the turbidity was actually caused by the leaked bacteria (Figure 3).

### 2.5. Statistical Analysis

All data were organized in a contingency table. A linear regression model (SPSS/PC Statistics 21 software; SPDD International BV, Gorinchem, the Netherlands) was used and leakage data were analysed statistically by a chi-square test. The level of significance was set at *p* < 0.05

## 3. Results

Table 2 shows the leakage between materials on the 7th and 45th days. A significant result was determined between the groups on the 7th day following repair. The group where perforations were created with size 2 burs and MTA was used as a repair material showed the lowest leakage with 57.1% of nonleaked samples compared to the other groups (*p* < 0.05).

## 4. Discussion

The goal of endodontic treatment is to eliminate bacteria and maintain a hermetic seal throughout the root canal system. From this point of view, hermetic seal is supposed to have an impact on clinical success in the long term; however, it is not possible to determine this parameter under clinical conditions.

Various methodologies have been described to measure microleakage such as dye penetration method, radio isotope tests, bacterial tests, electrochemical tests, nanoleakage tests, and liquid filtration methods. On the other hand, there is yet no consensus regarding the best methodology to be used for the assessment of leakage [7, 8].

It has been discussed that the dye liquid penetration [8] methods do not reflect clinical conditions due to insufficient molecular weight of the dye which prevents it to penetrate areas where bacteria are able to access [9].

Oliver and Abbott evaluated the success of the root canal materials in clinical conditions and concluded that dye penetration is an unreliable method for measuring leakage [10]. Comments and criticism on dye leakage studies resulted in bacteriological studies to become more popular due to their resemblance to clinical conditions. Therefore, bacteriological examination was selected as the methodology to assess leakage.

The experiments were concluded on the 50th day as all samples displayed leakage at the end of 45 days. No leakage was observed in the negative control group showing that the junction points, tube, or the silicon hose were adequately sealed.

Coronal leakage causes the microorganisms to infiltrate into the canal system and causes apical reactions. The contaminated pulp chamber may serve as a reservoir for bacteria resulting in failure of the endodontic treatment [11, 12]. Bacterial leakage studies were reported to be more accurate compared to clinical studies for their biological consistency [13–17].

On the other hand, utilization of only a single species of microorganism may be regarded as a disadvantage of the methodology considering the wide variety of microorganisms present in the root canal system. The reason for selecting a single species was to provide standardization between groups.* E faecalis *was chosen for contamination as it is generally regarded as the gold standard in the field of endodontology for its persistent characteristics [18–20].

*E. Faecalis *colonies were embedded in a specific agar to ensure that the leaked bacteria was* E. faecalis* and there was no contamination of the set-up.

The mixing type, time, and powder/liquid ratio are very important factors in the standardization of the materials. The capsulation method is used for amalgam, glass ionomer, chemical composite resins, zinc phosphate, and calcium hydroxide and enhances the standardization of the mixture of the materials. It decreases the air space between the materials and allows the powder to better penetrate into the liquid. The mixture of MTA with capsulation was reported to be a more efficient means of providing a homogeneous saturation of the material [21–24].

In the present study, Biodentine was mixed using an amalgamator according to the manufacturers' instructions. On the other hand, Original ProRoot MTA was mixed in a capsule as described by Nekoofar et al. (2010a) rather than the capsulated form of MTA which recently became commercially available, due to some differences in terms of ingredients [23].

MTA is a biocompatible material so it is advocated to be used as a repair material in orthograde and retrograde perforations.

One disadvantage of this popular material is the difficulty of manipulation and placement due to its consistency. As the MTA hydrates it releases calcium hydroxide and calcium silicate crystals. The crystals bind together and combine to form a network made up of pores. When the hydration increases, the microcanals in MTA decrease and hold the excess water. This causes MTA to set and enhance the sealing ability of the material. The smaller the perforation is, the better the sealing can be achieved.

In perforations of smaller magnitude, better sealing canal be obtained, resulting in a more successful sealing ability.

While an amalgamator was used during the mixing of MTA and Biodentine, IRM was prepared by hand mixing because that was the only option. Consequently, materials mixed using an amalgamator might result in the formation of a more homogeneous mixture and a better sealing ability. Furthermore, in case a base material was used for the materials tested, leakage would be much less due to the prevention of direct contamination. The results of the study might also be different in case composite resin restoration was placed onto the repair material.

## 5. Conclusions

Pro Root MTA was determined as the most successful in terms of preventing microleakage when compared with IRM and Biodentine. The diameter of the perforation was found to have an impact on microleakage and the preforation which is 2 mm in diameter exhibited less leakage compared to a 4 mm perforation. The amounts of microleakage increased by time in all materials.

## Figures and Tables

**Table 1:** The distribution of the teeth and repair materials among groups.

Group 1 size 2 bur *n* = 60	Group 1-MTA *n* = 20	Group 1-BIOD *n* = 20	Group 1-IRM *n* = 20	(+) Control *n* = 10

Group 2 size 4 bur *n* = 60	Group 2-MTA *n* = 20	Group 2-BIOD *n* = 20	Group 2-IRM *n* = 20	(−) Control size 2 bur *n* = 5 size 4 bur *n* = 5

**Table 2:** Showing the leakage between 7th and 45th days.

Perforation repair material	Perforation size number: 2 bur Number of leaked specimens				Perforation size number: 4 bur Number of leaked specimens			
	7 day	15 days	30 days	45 days	7 days	15 days	30 days	45 days
MTA	12	11	9	0	7	5	2	0
Biodent.	5	4	2	0	6	5	3	0
IRM	4	3	2	0	6	5	4	0

Total	21	18	13	0	19	15	9	0
